# Composite Polymer Electrolyte Based on PAN/TPU for Lithium-Ion Batteries Operating at Room Temperature

**DOI:** 10.3390/polym16233280

**Published:** 2024-11-25

**Authors:** Xuanan Lu, Jianguo Luo, Lingxiao Lan, Yujiang Wang, Xinghua Liang, Junming Li, Aijun Fu

**Affiliations:** 1 Guangxi Key Laboratory of Automobile Components and Vehicle Technology, Guangxi University of Science & Technology, Liuzhou 545006, China; a15007751851@163.com (X.L.); 18677215099@163.com (J.L.); llx2685062@163.com (L.L.); lxh304@126.com (X.L.); jmli@gxust.edu.cn (J.L.); 2 Guangxi Transportation Industry Key Laboratory of Vehicle-Road-Cloud Integrated Cooperation, Guangxi University of Science & Technology, Liuzhou 545006, China

**Keywords:** PAN, TPU, composite polymer electrolytes, lithium-ion battery

## Abstract

Lithium-ion batteries have garnered significant attention owing to their exceptional energy density, extended lifespan, rapid charging capabilities, eco-friendly characteristics, and extensive application potential. These remarkable features establish them as a critical focus for advancing next-generation battery technologies. However, the commonly used organic liquid electrolytes in batteries are explosive, volatile, and possess specific toxic properties, resulting in persistent safety concerns that remain to be addressed. Composite polymer electrolytes (CPEs) exhibit enhanced safety and stable electrochemical performance, emerging as one of the most promising alternatives. However, single polymers often need to meet the multifaceted performance requirements of batteries. In this study, a composite polymer electrolyte was prepared using solution casting, consisting of a blend of polyurethane (TPU) and polyacrylonitrile (PAN), along with the ceramic filler Li_1.3_Al_0.3_Ti_1.7_(PO_4_)_3_ (LATP) and lithium perchlorate (LiClO_4_). The optimal formulation, which included 40 wt% TPU, 60 wt% PAN, and 10 wt% LATP, exhibited a commendable ionic conductivity of 2.1 × 10^−4^ S cm^−1^, a lithium-ion transference number (t_Li_^+^) of 0.60, and notable electrochemical stability at 30 °C. The LiFePO_4_/Li battery assembled with this CPE demonstrated excellent cycling stability and rate capability at room temperature. It delivered a discharge specific capacity of 130 mAh g^−1^ at 1C. Under a charge–discharge rate of 0.2C, the battery achieved a discharge specific capacity of 168 mAh g^−1^, retaining 98% of its capacity after 100 cycles at 25 °C. Additionally, the CPE exhibited robust safety performance. Consequently, this composite polymer electrolyte holds significant promise for application in lithium-ion batteries.

## 1. Introduction

Lithium-ion batteries have ascended as the heralded third generation of energy storage technologies, succeeding the pioneering realms of nickel–cadmium and nickel–metal hydride batteries [1]. They are now widely used in consumer electronics, military equipment, and electric vehicles [2,3]. However, conventional lithium-ion batteries, such as electrolyte leakage and uncontrolled side reactions, still pose safety risks. These reactions can cause overheating, leading to the shrinkage of commercial Celgard separators, which can ultimately result in short circuits, fires, or explosions [4,5]. Hence, it is essential to devise battery systems that prioritize safety, which is distinguished by outstanding thermal stability and superior mechanical robustness [6,7].

In recent years, substituting traditional liquid electrolytes and separators with solid-state electrolytes has been recognized as the most efficient strategy. This replacement resolves the previously mentioned safety concerns and provides enhanced energy densities [8,9]. Solid-state electrolytes encompass inorganic solid electrolytes, polymer electrolytes, and composite polymer electrolytes (CPEs) [10]. Among these, inorganic solid electrolytes feature a lithium-ion transference number (t_Li_^+^) of about one [11] and high ionic conductivity (σ), such as 1.2 × 10^−2^ S cm^−1^ for sulfide electrolytes [12]. However, pure inorganic solid electrolytes suffer from poor interfacial contact, high interfacial impedance, and brittleness, which lead to performance degradation during operation [13]. These issues hinder their rapid development. On the other hand, polymer electrolytes like polyethylene oxide (PEO) [14], polyvinylidene fluoride (PVDF) [15], and polyacrylonitrile (PAN) [16] possess distinct advantages, such as ease of processing, good electrode contact, and suitability for flexible battery designs. However, they typically suffer from low ionic conductivity at room temperature, around 10^−6^~10^−5^ S cm^−1^, which seriously restricts their application in consumer electronics [17]. Additionally, their narrow voltage window, with a cutoff voltage below 4 V, prevents compatibility with high-voltage cathode materials like nickel–cobalt–manganese, making it challenging to meet the high-energy-density demands of future energy storage systems [18]. Changmin shi et al. proposed the design of CPEs using different polymer matrices and inorganic fillers in order to achieve good ionic conductivity and excellent mechanical deformability, which is one of the most popular synthesis methods [19]. Therefore, organic–inorganic composite polymer electrolytes combine the advantages of polymer electrolytes with inorganic solid electrolytes, which has become a hot spot in current research.

Polyacrylonitrile (PAN) exhibits excellent properties, including high thermal stability, high ionic conductivity, good compatibility with lithium electrodes, favorable electrolyte absorption, and the ability to minimize dendrite formation during charge and discharge cycles [20]. The cyano (C≡N) groups within PAN can interact with lithium ions and carbonyl (C=O) groups in ethylene carbonate (EC), enhancing its versatility for various applications [21]. However, significant interfacial passivation occurs between PAN-based gel polymer films and lithium electrodes. Additionally, the mechanical properties of PAN deteriorate with an increasing plasticizer content [22]. Consequently, PAN cannot serve as an independent membrane in lithium-ion batteries. PAN has been modified through copolymerization or blending with other materials to improve the mechanical stability.

Thermoplastic polyurethane (TPU) is an elastomer characterized by high tensile strength, high elasticity, and low crystallinity [23]. For lithium-ion battery membranes, superior mechanical properties, flexibility, and stiffness are crucial. TPU features a biphasic microstructure with soft and hard segments. The hard segments attach to the soft segments, maintaining spatial stability through π-π interactions and hydrogen bonding between urethane groups [24]. The soft segments, containing ether linkages, can support ionic liquids, facilitating the dissociation of alkali metal salts and thereby enhancing the ionic conductivity. The physical crosslinking of the soft segments ensures good film formation and robust mechanical properties [25]. TPU is widely used in various industrial applications due to its excellent physical properties, chemical resistance, wear resistance, good adhesion, and ease of processing.

Blending two or more polymers is recognized as an effective approach to enhancing the ionic conductivity and dimensional stability of polymer electrolytes [26]. The primary advantage of this method lies in its simplicity. By adjusting the proportions of the polymer components, electrolytes with combined advantages can be developed, improving the mechanical and electrical properties. Therefore, this study blends TPU and PAN to control the physical properties of the mixture, improve the chemical performance synergistically, and achieve a higher ionic conductivity.

Research has indicated that the amalgamation of inorganic solid electrolytes with polymer matrices effectively diminishes the crystallinity of the polymers while simultaneously enhancing the transport kinetics of lithium ions, thus facilitating a notable increase in the ionic conductivity. Li_1.3_Al_0.3_Ti_1.7_(PO_4_)_3_ (LATP) is a glass–ceramic material distinguished by its three-dimensional Sodium Superionic Conductor (NASICON)-type framework [27]. The channel dimensions within LATP approximate the diameter of lithium ions, thereby facilitating their effective migration throughout the material. Moreover, LATP is characterized by outstanding mechanical strength, exceptional chemical stability, superior ionic conductivity, and robust thermal stability at elevated temperatures, making it a significantly promising material for solid electrolyte applications [28].

In this work, we present composite polymer electrolytes consisting of LiClO_4_, PAN, TPU, and LATP, denoted as TPU/PAN/LATP-LiClO_4_ and labeled as TPU-X, where X represents the ratio of the TPU polymer mass to the total mass of the TPU and PAN. Using a blended TPU/PAN polymer matrix and LATP filler, TPU-40 achieves a high mechanical strength, excellent thermal stability, and favorable ionic conductivity. The LiFePO_4_|TPU-40|Li battery also demonstrates significantly enhanced rate performance and superior cycling performance. Furthermore, the battery operates safely even under mechanical folding, drilling, and cutting, highlighting its high level of safety.

## 2. Materials and Methods

### 2.1. Materials

Polyurethane (TPU; Macklin, Shanghai, China), Polyacrylonitrile (PAN; Macklin, Shanghai, China) powder, Potassium Perchlorate (LiClO_4_; Macklin, Shanghai, China), Li_1.3_Al_0.3_Ti_1.7_(PO_4_)_3_ (LATP; Macklin, China), N, N-Dimethylformamide (DMF; Macklin, China), LiFePO_4_ (LFP; Macklin, Shanghai, China), conductive carbon black (Super P; Macklin, Shanghai, China), and polyvinylidene fluoride (PVDF; Macklin, Shanghai, China). All of the materials were used directly without further processing. TPU and PAN were dried separately in a vacuum oven before use.

### 2.2. Preparation of TPU-X Electrolyte

**Figure 1:**
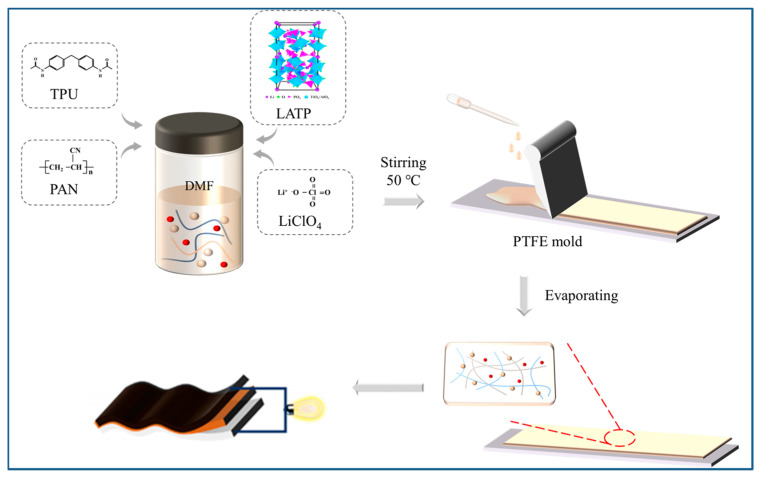
Flow chart of the electrolyte preparation.

TPU-X composite polymer electrolytes (CPEs) were prepared using the solution casting method (Figure 1). First, TPU and PAN powders were added to a DMF solvent with a total mass of 2 g of TPU and PAN, where the mass of TPU was X% of the total mass of TPU and PAN. The solution was stirred at 50 °C for 1 h, and then LiClO_4_ was added. An amount of 0.2 g of LATP was subsequently added and stirred for 3 h. Solutions of TPU-0, TPU-20, TPU-40, TPU-60, and TPU-80 were obtained, respectively. The homogeneously stirred solution was introduced into a polytetrafluoroethylene plate, and TPU-X GPE was obtained by drying it in a vacuum drying oven at 60 °C for 2 h. TPU-X CPE was cut into φ18 mm discs and put into a glove box for use.

### 2.3. Assembly of the Battery

To modulate the LFP slurry, LFP, PVDF, and Super P were mixed into N-Methyl-2-pyrrolidone (NMP) in a mass ratio of 8:1:1. The LFP slurry was applied to the aluminum foil with a spatula, and the LFP positive electrode sheet was vacuum-dried at 60 °C for 36 h. The cathode electrode sheet was cut into disks with a diameter of 14 mm prior to use. A discharge/charge rate of 1C corresponded to a specific current of 170 mA g^−1^. Standard CR2025 coin cells were assembled in an argon-filled glove box. A lithium metal disk (0.6 mm thickness; 16 mm diameter) was used as the anode. All of the cells were assembled in an argon-filled glove box.

### 2.4. Methods of Characterization

#### 2.4.1. X-Ray Diffraction Analysis

The crystallization properties of the TPU-X and other materials were studied using an X-ray diffractometer (XRD; DX-2700, Dandong, China). In this experiment, the operating voltage of the XRD was set at 40 kV, with a scanning speed of 0.2° s^−1^ and a scanning range of 2θ from 10° to 90°. When measuring the electrolyte membrane, the membrane was fixed on the sample holder, ensuring a smooth surface without any noticeable scratches or stains. For measuring the LATP, PAN, and TPU powders, they were first ground into a fine powder using a mortar and pestle. The powder was then sieved through a mesh screen, and the sieved powder was placed on a sample holder specifically designed for XRD tests. The powder was then compacted and leveled.

#### 2.4.2. Scanning Electron Microscopy Analysis

The surface and cross-section of TPU-X were observed and analyzed using a scanning electron microscope (SEM; Phenom spectra G2, Shanghai, China), followed by EDS spectral analysis. In this experiment, the test film was first cut into small pieces, and conductive tape was adhered to the sample stage before placing the test film. A gold coating was then applied to the surface of the film for 120 s to render the previously non-conductive film surface conductive. Finally, the sample stage was inserted into the testing instrument for the analysis.

#### 2.4.3. Fourier Infrared Spectral Analysis

Fourier Transform Infrared Spectroscopy (FTIR; Spectrum 100, PerkinElmer, Shelton, CT, USA) was employed to determine the chemical bonds of TPU-0, TPU-40, TPU, and PAN within the range of 4000–400 cm^−1^. This test utilized the ATR method, during which the electrolyte membrane was cut into small sizes suitable for the infrared spectrometer sample holder. The membrane was then directly placed on the ATR crystal and gently pressed to ensure good contact.

#### 2.4.4. Thermogravimetric Analysis

The thermal stability of thermoplastic polyurethane-X was investigated using thermogravimetric analysis (HTG, HTG-2, and HENVEN) within a temperature range from 30 °C to 800 °C, with a heating rate of 10 °C min^−1^ under nitrogen atmosphere. Before testing, 10 mg of the electrolyte was cut and dried in a low-temperature oven to remove moisture from the sample.

### 2.5. Electrochemical Performance Test

The assembled SS|CPE|SS symmetric cells were tested for impedance at room temperature. The ionic conductivity was calculated by Equation (1). The frequency range was set to 0.01~10^6^ Hz. The following equation calculated the ionic conductivity:

(1) $$\sigma =\frac{L}{R\cdot S}$$

Here, *L* represents the thickness of TPU-X, *Rg* denotes the bulk resistance, and *S* indicates the geometric contact area between TPU-X and the encapsulated stainless steel electrode.

Electrochemical impedance spectroscopy (EIS) and the timed current method were used to determine the lithium-ion mobility number (t*_Li_*^+^). A polarization voltage (Δ*V*) of 10 mV was applied to the symmetric cell (Li|TPU-X|Li). The initial and steady-state values of the current were then measured separately. Then, combining the interface resistance of the initial and steady-state values, t*_Li_*^+^ was calculated by Equation (2) as follows:

(2) $$t_{Li}^{+}=\frac{I_{s}\left(\Delta V-R_{0}I_{0}\right)}{I_{0}\left(\Delta V-R_{s}I_{s}\right)}$$

where *I*_0_ is the initial current, *R*_0_ is the interface resistance before polarization, *I_s_* is the steady-state value, *R_s_* is the interface resistance after polarization, and Δ*V* is 10 mV.

The electrochemical stability of the TPU-X CPE was measured using linear scanning voltammetry (LSV) at potentials of 2–6 V (Li^+^/Li) with a scan rate of 5 mV/s. All of the above chemical measurements were carried out on an electrochemical workstation (DH7000, Donghua, Jingjiang, China). The assembled LFP|TPU-X|Li batteries were charged and discharged using the NEWARE Battery Test System (BTS-4000, Shenzhen, China) to test the assembled batteries’ charging/discharging curves, multiplication performance, and cycling performance.

## 3. Results

### 3.1. Li^+^ Conduction in Composite Polymer Electrolytes (CPEs)

**Figure 2:**
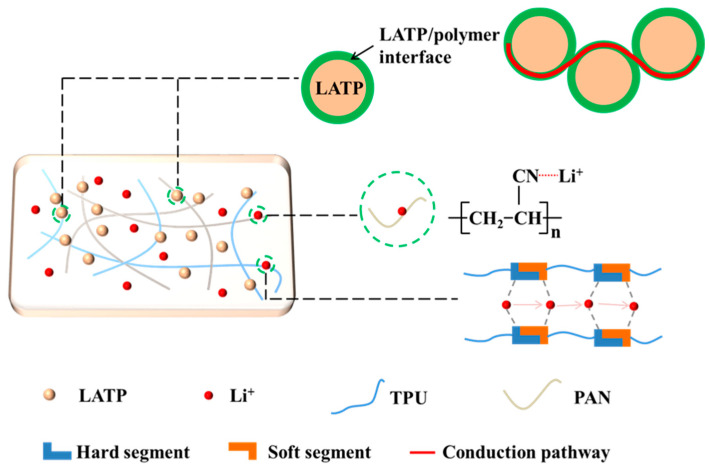
Diagram of lithium-ion transport pathways in CPEs.

In Figure 2, the transport mechanism of lithium ions within the electrolyte is depicted. The inclusion of active fillers promotes the establishment of space charge zones at the polymer/LATP juncture, thereby fostering the clustering of lithium ions on one side of the interface. Upon the interconnection of space charge regions formed within discrete nanoparticles, a rapid lithium-ion transport pathway is established, resulting in a substantial enhancement of the ionic conductivity [29]. The C≡N polar group in PAN can coordinate with Li and dissociate the Li salt, which plays a key role in Li transport along the polymer molecular chain [30]. The -C-O-C- in the soft chain segments of TPUs and -C=O and -N-H in the hard chain segments participate in lithium-ion transport during electrochemical cycling [31]. The hard/soft phase of TPUs serves a dual function in polymer electrolytes, giving the electrolyte a high strength, good film-forming ability, and facilitating lithium-ion transport. In conclusion, the electrolyte blended with TPU and PAN has a good lithium-ion transport channel, which can be effective for lithium-ion transport.

### 3.2. Material Properties

**Figure 3:**
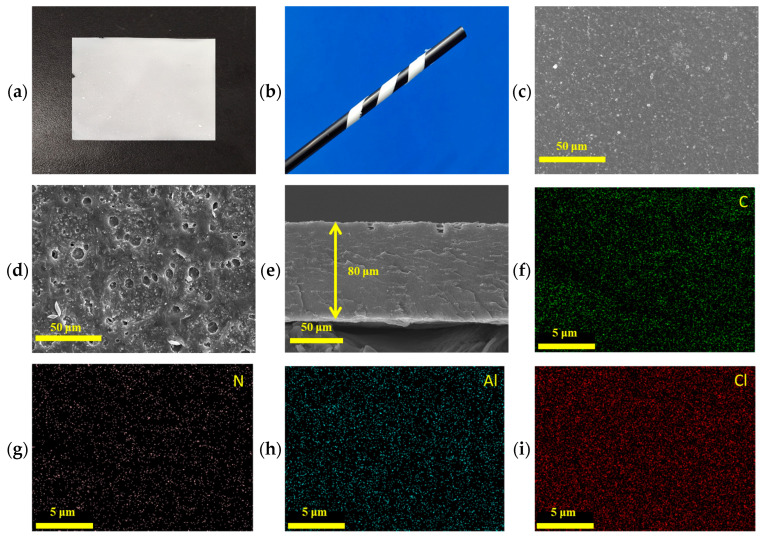
(**a**) Digital photograph of the surface of the TPU-40 electrolyte membrane; (**b**) Image of the wrapped TPU-40 electrolyte membrane to demonstrate its flexibility; (**c**) SEM image of the cross-section of the TPU-40 electrolyte membrane; (**d**) SEM image of the surface of the TPU-40 electrolyte membrane; (**e**) SEM image of the surface of the TPU-0 electrolyte membrane; (**f**–**i**) EDX mapping images of the cross-sectional SEM image of the TPU-40 electrolyte membrane: (**f**) carbon is represented in green, (**g**) nitrogen in purple, (**h**) aluminum in blue, and (**i**) chlorine in red.

Figure 3a presents an image of the flexible TPU-40 membrane, while Figure 3b demonstrates its exceptional flexibility and bending capability, allowing it to be twisted into any shape. The cross-sectional view of the composite polymer electrolyte (CPE) membrane shown in Figure 3c indicates that it forms continuously and uniformly without pores or aggregation. The thickness of this CPE membrane is approximately 80 µm. Figure 3d,e display the surface views of the TPU-40 and TPU-0 GPEs, respectively. It is evident from the figures that the surface of TPU-40 is rougher and features pores. This phenomenon can be attributed to the following two main reasons: (1) the differing solubility of TPU and PAN affects the evaporation rate of the solvent, leading to the formation of irregular pore structures on the film surface; (2) TPU acts as a plasticizer, enhancing the flow ability of PAN and resulting in the creation of more voids during the membrane formation process. These pores and the surface roughness contribute to a larger specific surface area, facilitating ion transport and potentially enhancing the ionic conductivity. Figure 3f–i illustrate the EDS analysis of the TPU-40 electrolyte, revealing a uniform distribution of various elements. The overall distribution of these elements suggests that the LATP filler and LiClO_4_ are evenly dispersed within the polymer matrix, potentially enhancing the electrochemical performance of the CPE [32].

**Figure 4:**
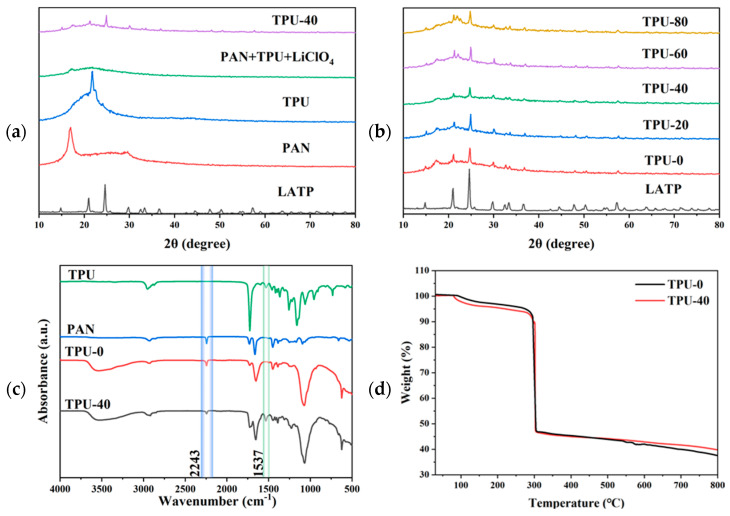
(**a**) XRD patterns of the LATP, PAN, and TPU powders, PAN+TPU+LiClO_4_ membranes, and TPU-40 membranes; (**b**) XRD patterns of the LATP and TPU-X membranes; (**c**) Fourier Transform Infrared (FT−IR) spectra of the PAN, TPU, TPU-0, and TPU-40 membranes; (**d**) TG curves of the TPU-0 and TPU-40 electrolyte membranes.

Figure 4a displays the XRD patterns of the LATP, PAN, TPU powders, PAN + TPU + LiClO_4_ membranes, and TPU-40 membranes. No characteristic peaks of LiClO_4_ were observed in the PAN + TPU + LiClO_4_ electrolyte, indicating that LiClO_4_ was completely dissolved. As can be seen from Figure 4b, with an increase in the TPU content, the diffraction peak intensity of TPU around 20° in the TPU-X electrolyte membrane gradually rises. In contrast to the TPU-0 membrane, the diffraction peaks of the TPU-20, 40, 60, and 80 membranes are broader and less intense, indicating that the electrolyte formed by the blending of TPU and PAN has lower crystallinity [33]. This reduced crystallinity is beneficial for lithium-ion transport. Therefore, we expect the ionic conductivity of CPE to increase due to the decrease in the crystalline phase content of TPU/PAN. In addition, the crystallization peaks of LATP in CPE were almost the same as those of the pure LATP powder. Therefore, no degradation of the LATP powder occurs in the CPE, which still maintains its crystalline structure.

Figure 4c presents the Fourier Transform Infrared spectra of the TPU-0 and TPU-40 membranes. In the FT-IR spectrum of the TPU-40 membrane, the peaks around 1537 cm^−1^ correspond to the –C(=O) –N– amide group of the TPU, respectively [34]. The peak near 2243 cm^−1^ corresponds to the stretching vibration of the –C≡N group in PAN [35]. In the TPU-40 membrane, the intensity of this characteristic peak is diminished. This reduction may be attributed to the blending process, where the introduction of TPU potentially decreases the relative content of PAN, subsequently leading to a lower concentration of the –C≡N groups and weakening the absorption intensity of this peak. Compared to the FT-IR spectra of TPU and PAN powders, the FT-IR spectra of the TPU-0 and TPU-40 membranes do not exhibit any new chemical bonds. This indicates that the blending of TPU and PAN occurs purely as a physical mixture without any chemical reactions taking place.

In order to evaluate the thermal stability, the thermogravimetric analysis (TG) curve for the CPE membrane is presented in Figure 4d. TPU-0 and TPU-40 showed 1–2% mass loss at 30–100 °C due to the evaporation of water and DMF. At 100–150 °C, there is about a 5% weight loss, attributed to water vapor loss from the hygroscopic CPE membrane and the partial decomposition of DMF in the electrolyte [36]. Thermal decomposition of the CPE membrane commences at 280 °C, mainly due to the decomposition processes of TPU, PAN, and LiClO_4_ [37]. The exceptional thermal stability attained herein underscores the suitability of the composite electrolyte membrane for fulfilling the operational criteria of lithium-ion batteries.

**Figure 5:**
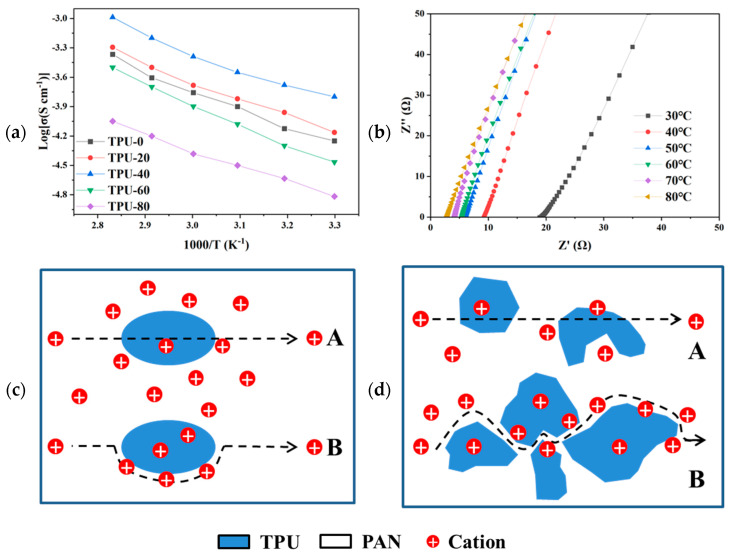
(**a**) Temperature dependence of ionic conductivity for the TPU-X electrolyte membranes; (**b**) Nyquist plots of TPU-40 from 30 to 80 °C. Schematic representation of two possible lithium-ion transferring routes. Path A: the ions transfer through different phases; Path B: the ions transfer around the interface between different phases. (**c**) The TPU/PAN electrolyte with a low TPU concentration and (**d**) with a high TPU concentration.

Ionic conductivity is a crucial factor that influences the electrochemical performance of polymer electrolytes. Figure 5a illustrates the ionic conductivity of five types of electrolyte membranes within the temperature range from 30 to 80 °C. The TPU-40 membrane exhibits the highest ionic conductivity at 30 °C, measuring 2.1 × 10^−4^ S cm^−1^. In contrast, the ionic conductivity of the TPU-0 electrolyte was only 6.8 × 10^−5^ S cm^−1^. The high ionic conductivity of the electrolyte is due to the fact that lithium ions can be rapidly conducted at the interface between the inorganic filler LATP and the polymer in the composite solid-state electrolyte. There is a strong affinity between Lewis acid centers on the surface of ceramic particles and ClO_4_^−^ in lithium salts, which helps to separate Li^+^ and ClO_4_^−^ ion pairs to increase the concentration of free Li^+^ ions [32]. The higher ionic conductivity of the TPU-40 electrolyte helps to increase the charging and discharging rate of the battery, enabling the battery to complete the charging and discharging process in a short period of time. Additionally, the activation energies for the TPU-X electrolyte membranes were calculated using the Arrhenius equation. The activation energies are as follows: TPU-0 at 0.161 eV, TPU-20 at 0.142 eV, TPU-40 at 0.139 eV, TPU-60 at 0.179 eV, and TPU-80 at 0.161 eV. The TPU-40 electrolyte membrane has the lowest activation energy, which facilitates faster ion movement through the membrane, thereby enhancing the overall ionic conductivity. Figure 5b presents the Nyquist plots of the TPU-40 membrane at various temperatures. As the temperature increases, the bulk resistance of the membrane decreases, demonstrating the significant impact of temperature on the ionic conductivity of the battery.

The ionic conductivity of polymer-blend electrolytes is substantially influenced by the compatibility and interfacial adhesion between the constituent phases. In polymer electrolytes characterized by inadequate interfacial adhesion, the presence of distinct boundaries creates discontinuities, impeding ion transfer across the phase interfaces [38]. Figure 5c,d elucidate the divergent ion transfer mechanisms operative at the phase interface. In the context of superior interfacial adhesion, ions traverse the interface via pathway A; conversely, in the presence of deficient interfacial adhesion, ions circumvent the interface through pathway B. The latter route significantly increases the path length for ion movement between the electrodes and leads to high resistance at the interface. The SEM image of the TPU/PAN electrolyte shows that the TPU/PAN electrolyte has good interfacial adhesion. In this case, path A dominates, and therefore their ionic conductivity increases with an increasing PAN concentration. The prodigious ionic conductivity exhibited by the TPU-40 electrolyte can be attributed to a dual mechanism: firstly, the intrinsic high ionic conductivity of the PAN electrolyte augments the proportion of the high-ionic-conductivity component within the blended electrolyte; secondly, the presence of LiClO_4_ enhances the compatibility between TPU and PAN, thereby fortifying the interfacial adhesion. This advantageous adhesion promotes the efficient migration of lithium ions across the phase boundaries, avoiding the requirement for sidestepping the interfacial limitations. Upon attaining a certain loading, the TPU induces a decline in interfacial adhesion at specific locations within the electrolyte, prompting a switch wherein certain ions are no longer transferred via pathway A but instead commence migration through pathway B.

**Figure 6:**
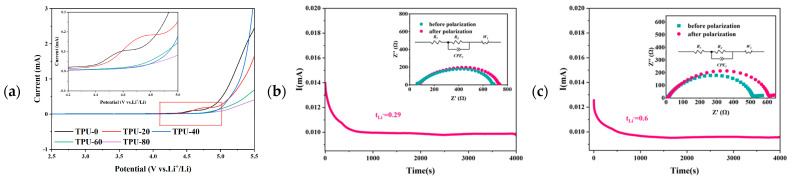
(**a**) LSV curves of TPU-X electrolyte membranes; (**b**) Initial and steady−state impedance and polarization curves for the TPU-0 electrolyte, with an inset showing the corresponding EIS plots before and after polarization; (**c**) Initial and steady−state impedance and polarization curves for the TPU-40 electrolyte, with an inset showing the corresponding EIS plots before and after polarization.

Electrochemical stability is an essential factor affecting the practical application of lithium-ion batteries. The linear sweep voltammetry curve of the TPU-X electrolyte membrane, shown in Figure 6a, is employed to evaluate the oxidative stability of the electrolyte membrane. The current of the TPU-X electrolyte membrane maintains a consistent level between 2.5 and 4.3 V. Remarkably, the TPU-40 electrolyte displays no significant increase in current within the range of 2.5 to 4.5 V, thereby signifying its superior oxidative stability.

From Figure 6b,c, it can be observed that the lithium-ion transference number (t_Li_^+^) for TPU-40 is measured at 0.60, which is significantly higher than that of TPU-0 (0.29). The high migration number helps to reduce the resistance and polarization phenomenon in the ion transport process, which significantly reduces the internal resistance of the battery and improves the charging and discharging efficiency. In addition, the high mobility number also makes the lithium ions evenly distributed in the electrolyte, which reduces the phenomenon of localized over-concentration or under-concentration on the electrode surface. This uniform distribution effectively reduces the structural changes and capacity degradation of the electrode material, which, in turn, improves the cycling stability of the battery. Therefore, the TPU-40 electrolyte film has a higher Li-ion migration number and is more likely to achieve stable battery performance.

### 3.3. Mechanical and Thermal Stability of Composite Polymer Electrolytes (CPEs)

**Figure 7:**
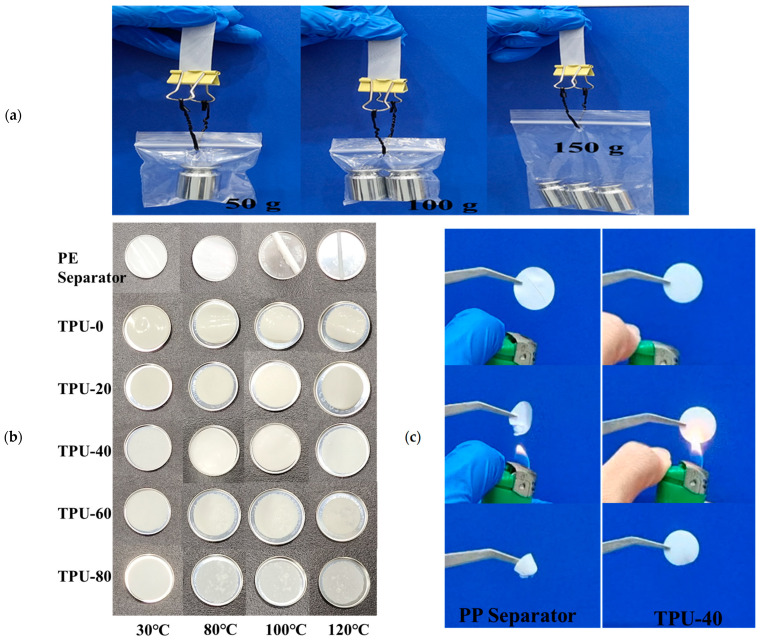
(**a**) Optical images demonstrating the mechanical strength of the TPU-40 membrane; (**b**) Thermal properties of various electrolytes and polyethylene (PE) separators; (**c**) Flame retardancy tests of the Polypropylene Battery Separator (PP Separator) and TPU-40 electrolyte membrane.

In order to assess the mechanical performance of the electrolyte membrane, a rectangular TPU-40 membrane with dimensions of 1 × 8.0 cm^2^ was exposed to varying loads. As depicted in Figure 7a, this membrane demonstrated the capability to support weights of 50, 100, and 150 g, respectively, without exhibiting any signs of rupture.

The TPU-X electrolyte membrane and polyethylene (PE) separator were then subjected to various temperature conditions to evaluate their thermal stability. The PE separators exhibits no significant changes after 1 h at 80 °C. However, significant curling occurs after exposure to 100 °C and 120 °C, which increases the risk of contact between the positive and negative electrodes. This curling phenomenon is unfavorable for the safe use of batteries. For the TPU-60 electrolyte, bubbles were observed at 120 °C; for the TPU-80 electrolyte membrane, bubbles appeared at 80 °C. This phenomenon likely results from the softening or melting of TPU at high temperatures, leading to the formation of bubbles within the material or the expansion of pre-existing bubbles. After heating the TPU-0 electrolyte at 80 °C for 1 h, it began to bend, with the degree of bending progressively increasing as the temperature rose. In contrast, the TPU-20 electrolyte exhibited bending and contraction at 120 °C. This may be due to the inherent high-thermal-shrink ability of the PAN material, which significantly contracts at elevated temperatures, causing the electrolyte surface to wrinkle. The TPU-40 electrolyte membrane exhibited no significant changes across various temperatures, demonstrating its excellent thermal stability and safety. The stability of the TPU-40 electrolyte may be attributed to the close proportions of TPU and PAN, which can lead to the formation of a more stable blend, thereby enhancing the overall thermal stability of the material. Consequently, the blending of TPU and PAN can effectively improve the thermal stability of the electrolyte membrane.

In the following step, we compared the flame retardancy of the PP separator and the TPU-40 electrolyte membrane. As shown in Figure 7c, the PP separator begins to shrink when it approaches a lighter. Upon contact with the flame, the PP separator ignites rapidly and continues to burn even after being removed from the flame. Following combustion, the electrolyte exhibits significant shrinkage, which poses safety risks for the battery. In contrast, when the ignition source is removed, the TPU-40 electrolyte membrane extinguishes immediately after leaving the flame. This indicates that TPU-40 demonstrates excellent flame-retardant properties.

### 3.4. Electrochemical Performance Characterizations

**Figure 8:**
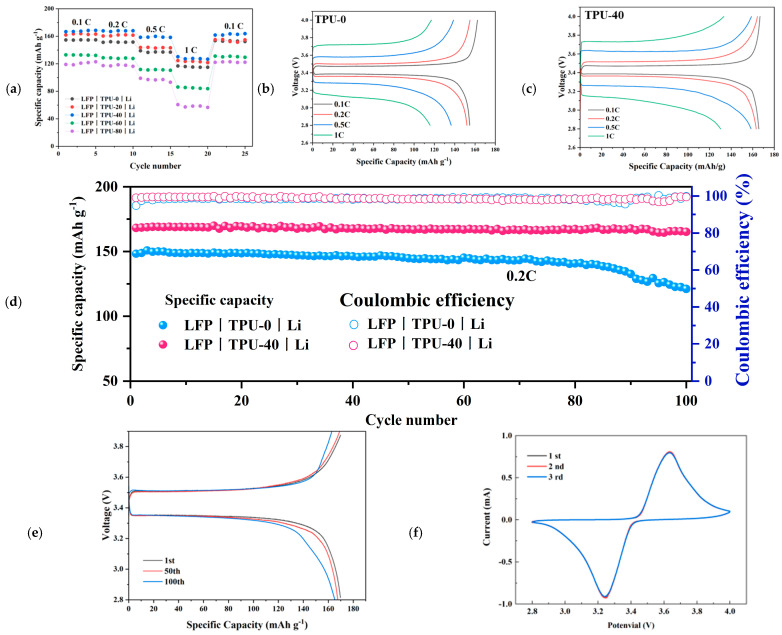
(**a**) Rate capability of the LFP|TPU-X|Li battery; (**b**) Voltage−discharge specific capacity graph of the LFP|TPU-0|Li battery at different multiplication rates; (**c**) Voltage−discharge specific capacity graph of the LFP|TPU-40|Li battery at different multiplication rates; (**d**) Cycling graphs of the LFP|TPU-0|Li and LFP|TPU-40|Li batteries at 25 °C; (**e**) Voltage−discharge specific capacity graph of the LFP|TPU-40|Li battery at different numbers of cycles; (**f**) Cyclic voltammetry of the LFP|TPU-40|Li battery.

The assembly of the LFP|TPU-X|Li battery was conducted to evaluate the electrochemical performance of the composite polymer electrolyte within the battery. The rate performance of all LFP|TPU-X|Li batteries was investigated. The rate performance was evaluated by varying the current density from 0.1 to 1C. As shown in Figure 8a, the LFP|TPU-40|Li cell exhibited the best rate capability, delivering discharge specific capacities of 168, 167, 158, and 130 mAh g^−1^ at current densities of 0.1C, 0.2C, 0.5C, and 1C, respectively. Figure 8b,c present the charge and discharge profiles of the LFP|TPU-0|Li and LFP|TPU-40|Li cells at various rates, highlighting the lithium insertion/extraction dynamics of the battery. Compared with other polymer electrolyte materials, the multiplicity performance of the TPU-40 electrolyte is higher than the rate performance of most of the batteries reported in the literature. Detailed preparation information is listed in Table 1, which shows that the electrolyte prepared by this scheme has good rate performance. The battery exhibits significant polarization at 0.5C and 1C currents, mainly attributable to the ion conductivity and interfacial characteristics of the solid electrolyte. First, the lower ion conductivity of the electrolyte results in a rate of ion transport that lags behind the electron transport rate under high current conditions, leading to charge buildup at the electrode–electrolyte interface and a consequent substantial voltage drop and increase in polarization. Secondly, the interfacial contact between the electrolyte and the electrode is generally not as effective as that with liquid electrolytes, resulting in higher contact resistance, which further exacerbates the polarization phenomenon.

Additionally, the LFP|TPU-40|Li lithium battery maintains a high specific capacity after 100 cycles at a 0.2C rate. At 0.2C, the initial discharge specific capacity of the LFP|TPU-40|Li battery is 168 mAh g^−1^. Figure 8d presents partial charge–discharge curves for the LFP|TPU-40|Li lithium-ion battery at 0.2C. As observed, the discharge specific capacity curve almost perfectly aligns with the charge specific capacity curve, indicating that the battery’s Coulombic efficiency is close to 100%. The voltage plateau of the LFP|TPU-40|Li lithium-ion battery remains relatively stable across different cycles. After the 1st, 50th, and 100th cycles, the discharge specific capacities are 168, 167, and 165 mAh g^−1^, respectively. After 100 cycles, the capacity retention of the LFP|TPU-40|Li battery is 98%. In contrast, the LFP|TPU-0|Li battery exhibits discharge specific capacities of 148, 144, and 121 mAh g^−1^ after the 1st, 50th, and 100th cycles. After 100 cycles, the capacity retention for the LFP|TPU-0|Li battery is only 82%. These results confirm that the LFP|TPU-40|Li lithium-ion battery exhibits an excellent rate capability and cycle stability.

The capacity of the LFP/TPU-0/Li cell decreases after about 80 cycles, mainly due to the reaction of C≡N in the PAN with lithium. While the weak interaction between the polymer (PAN) and the lithium salt (LiClO_4_) does provide a greater degree of freedom for lithium ions to move through the polymer matrix, thereby widening the electrochemical stability window and allowing the electrolyte to operate over a wider range of potentials, this weak interaction also has a number of negative effects, ultimately leading to a drop in battery performance around the 80th cycle. Firstly, the weak interaction implies the limited inhibition capacity of the electrolyte against side reactions. During the long-term operation of the battery, the C≡N bonds in PAN may react with the lithium metal anode, leading to the decomposition of PAN and the generation of side products such as C=N and C-N. These side products can disrupt the interface between the electrode and the electrolyte, resulting in uneven lithium-ion flux, thereby increasing the interface resistance and reducing the conductivity of lithium ions. Additionally, these reactions may also promote the formation of lithium dendrites and the accumulation of side-reaction products, further blocking the ion channels and increasing the internal resistance of the battery, ultimately causing a significant drop in battery capacity and cycling performance. Secondly, the weak interaction also leads to poor interface stability between the electrolyte and the electrode. During the battery cycling process, changes in the electrode volume can cause the deterioration of the electrolyte interface, further increasing the interface resistance and affecting the long-term cycling stability of the battery. In summary, although the weak interaction between the PAN and LiClO_4_ initially provides a broader electrochemical stability window, over time, and with increased cycling, issues such as the insufficient inhibition of side reactions and poor interface stability become more pronounced, ultimately leading to a significant degradation in the battery performance around the 80th cycle. While TPU has good flexibility and can better adapt to the infinite volume expansion of lithium metal, the excellent elastic modulus of TPU can effectively inhibit the growth of lithium dendrites [44,45]. Therefore, the LFP|TPU-40|Li Li-ion battery has better cycle stability than the LFP|TPU-0|Li Li-ion battery.

Figure 8f shows the cyclic voltammetry profile of the LFP|TPU-40|Li cell with oxidation peaks at 3.65 V and reduction peaks at 3.25 V at a scan rate of 0.2 mV s^−1^. The good overlap of the three cycles suggests that the cell possesses excellent reversibility in the redox reaction and high cycling stability, which is superior to those reported in the literature [43].

**Figure 9:**
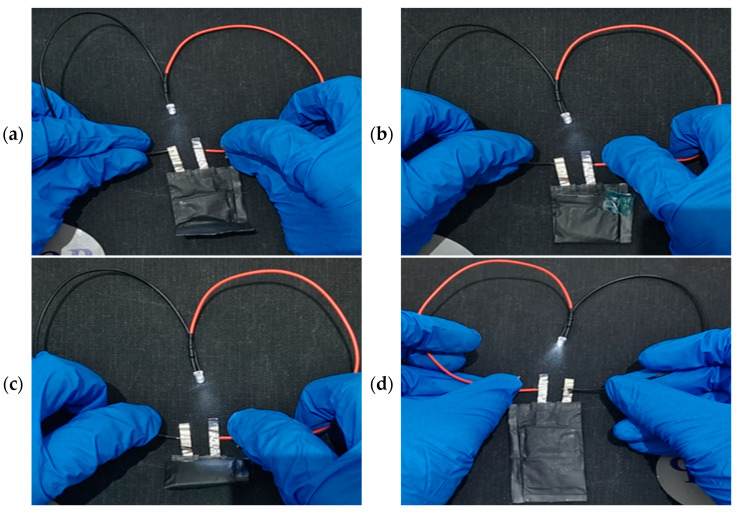
Digital photomicrograph of the LFP|TPU-40|lithium battery powering the LED under severe operating conditions. The operating conditions are (**a**) 90° folding, (**b**) 180° folding, (**c**) bending, and (**d**) restoring the battery to its original state.

The digital images presented in Figure 9 illustrate that the LFP|PPPL-10|lithium pouch cell retains the capacity to illuminate the LED device after undergoing (a) 90° folding, (b) 180° folding, and (c) bending, and the battery can still light up the LED after restoring the original state. Remarkably, no instances of short-circuiting were detected, thereby accentuating its robust safety features, which are anticipated to fulfill the stringent requirements of flexible and wearable technologies.

## 4. Conclusions

In this work, a composite polymer electrolyte was synthesized by incorporating a TPU/ PAN blend, along with the ceramic filler LATP and the electrolyte additive LiClO_4_. Compared to the pure PAN-based CPE, the TPU-40 electrolyte membrane exhibits a significantly higher ionic conductivity of 2.1 × 10^−4^ S cm^−1^, as well as enhanced mechanical strength, improved thermal stability, better oxidative stability, and a more significant lithium-ion transference number (t_Li_^+^ = 0.60). Additionally, substantial improvements in the rate performance and cycling stability have been demonstrated. The battery exhibits a discharge specific capacity of 130 mAh g^−1^ at a rate of 1C. Under a charge–discharge rate of 0.2C, the battery achieves a discharge specific capacity of 168 mAh g^−1^, with a capacity retention of 98% after 100 cycles at 25 °C. The self-extinguishing properties of the TPU-40 electrolyte, combined with its high mechanical and thermal stability, ensure safe operation even under bending conditions, such as 90° folding and 180° folding, highlighting its superior safety features. This work aims to provide guiding principles for designing high-performance composite polymer electrolytes and to inspire further research and applications in this field.

## Figures and Tables

**Table 1:** Comparison of the rate performance of the LFP|TPU-40|Li battery with other reported batteries.

Name of Electrolyte	0.1C (mAh g^−1^)	0.2C (mAh g^−1^)	0.5C (mAh g^−1^)	1C (mAh g^−1^)	Ref.
PAN/TPU/SHNT-3wt% ^1^	/	169	145	129	[39]
PAN/PO3TFSI ^2^/LiTFSI ^3^	155	135	118.7	96	[40]
TPU/PEO ^4^	/	140	131	112	[41]
PAN-HCP@UF ^5^	142	130	117	102	[42]
TSE-3 ^6^	151	143	129	116	[31]
PEO TPU 3:1 LLZO ^7^ 10%	170	161	144	115	[26]
The Zn|LiOH|FePO_4_ ^8^ battery			65 (0.5 mA/cm^2^)		[43]
TPU-40	168	167	158	130	This work

^1^ Surface-modified halloysite nanotube (SHNT). ^2^ 1,4-Diazabicyclo octane (DABCO) and diethylene glycol bis(2-chloroethyl)ether having bis(trifluoromethanesulfonyl) imide (TFSI−) as the anion (PO3TFSI, where ‘P’ indicates the oligomer and ‘O3’ denotes the 3 oxyethylene units) (PO3TFSI). ^3^ Lithium bis (trifluoromethanesulfonyl)imide salt (LiTFSI). ^4^ Poly(ethylene oxide) (PEO). ^5^ Thermal-response clothes for hexachlorophosphazene (HCP) additives by the microcapsule technique with urea–formaldehyde (UF) resin as the shell. HCP@UF successfully combines with polyacrylonitrile (PAN) by hydrogen bonds to form PAN–HCP@UF as the flame-retardant solid polymer electrolyte. ^6^ TSE was formed by introducing LiTFSI into a thermoplastic polyurethane (TPU). The solid electrolyte prepared when the mass ratio of LiTFSI to TPU was 1:1 was named TSE-3. ^7^ Li_7_La_3_Zr_2_O_12_ (LLZO). ^8^ Amorphous iron phosphate (FePO_4_).

## Data Availability

Data are contained within the article.
